# An Allergic Reaction to Henna Used in a Traditional Painting Ceremony

**DOI:** 10.4269/ajtmh.15-0833

**Published:** 2016-05-04

**Authors:** Yulia Treister-Goltzman, Eiman Egbaria, Roni Peleg

**Affiliations:** Department of Family Medicine, Siaal Research Center for Family Practice and Primary Care, Faculty of Health Sciences, Ben-Gurion University of the Negev, Beer-Sheva, Israel; Clalit Health Services, Southern District, Israel

**Figure 1.**
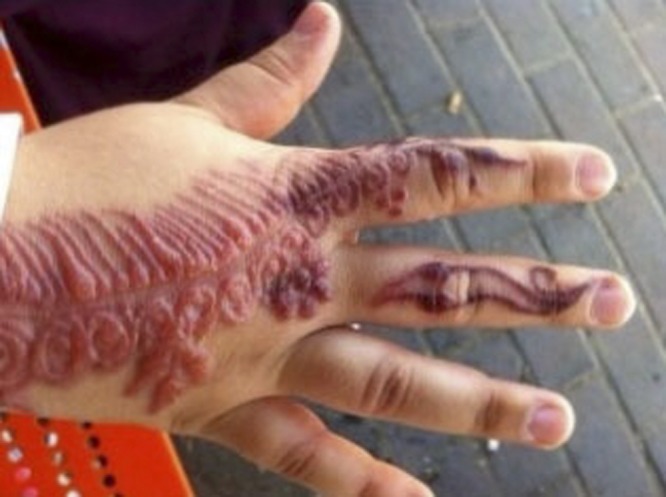
An allergic reaction to traditional henna.

A 22-year-old female patient, who was born in India and immigrated to Israel, presented with itching and a raised rash on her right hand (Figure 1

). The rash appeared a few days after the traditional henna ceremony, which was held on the day before her wedding. The patient is a healthy woman with no personal or family history of allergy or other atopic reactions. “Henna” painting is an ancient ceremony that prevails in all eastern communities and has been adopted by parts of the eastern Jewish communities. In the ceremony, a red-orange paste, which is produced from the henna plant (*Lawsonia inermis*), is spread on the bride's and the groom's hand as a blessing for prosperity and as a sign of good luck. The henna plant grows in the hot climate of northern Africa and southwest Asia, so its use is very prevalent in those regions.^1^ In the community of Indian Jews, the mother of the bride traditionally rubs the henna paste onto the fingers of the right hand where the wedding ring is to be worn.

It takes several hours after application of the paste until the red-brown shade materializes. Pure henna is a relatively safe product and allergic reactions to it are rare. The modern technique used to obtain a darker shade more quickly is to add *p*-phenylenediamine.^2^,^3^ Black henna tattoos induce contact allergy to its ingredient *p*-phenylenediamine at an estimated frequency of 2.5%.^4^ The classic allergic reaction to *p*-phenylenediamine is a type IV delayed-type hypersensitivity (DTH) reaction, but an acute life-threatening type I reaction has also been described.^1^ The manifestation of allergic dermatitis to *p*-phenylenediamine varies in severity from an intensely itchy erythematous, blistering eruption to painful, itchy exudative bullous eruptions, swelling, or renal collapse and failure.^5^ There have been reports of tattoo-induced chronic psoriasis on the tattoo sites despite treatment.^5^

The phenomenon reported in our patient is likely a type IV DTH response after haptenylation of host proteins. The patient was treated with topical steroid ointment with improvement in the itching and erythema.

Physicians who work in tropical regions or who treat immigrants from these regions should recognize and know the spectrum of traditional customs that can cause diseases and various skin problems.
